# Mucinous cystadenocarcinoma of breast in a 69-year-old woman with positive hormone receptors, the first case reported

**DOI:** 10.22088/cjim.12.0.444

**Published:** 2021

**Authors:** Ghodsieh kamrani, Novin Nikbakhsh, Akarm Hosseini, Hossein Ghorbani, Niloufar Arefisigaroudi, Ali Davarian

**Affiliations:** 1 Department of Pathology, Cancer Research Institute, Babol University of Medical Science, Babol , Iran; 2 Department of surgery, Cancer Research Institute, Babol University of Medical Sciences, Babol , Iran; 3 Department of Pathology, School of medicine, Babol University of Medical Sciences, Babol, Iran; 4 Ischemic Disorders Research Center, Golestan University of Medical Science, Gorgan, Iran

**Keywords:** Breast cancer, Mucinous cystadenocarcinoma, Estrogen receptor, Progesterone receptor

## Abstract

**Background:**

Mucinous cystadenocarcinoma is a relatively uncommon histological subtype of breast cancer that is a cystic form of papillary mucinous carcinoma.  It is regularly negative for estrogen and progesterone receptors and it is most often diagnosed in older than 55-60 years old. The incidence of breast mucinous cystadenocarcinoma is about 1-6% of primary breast cancers. Here, we present a case of breast mucinous cystadenocarcinoma of left breast in a 69-year-old female which is positive for estrogen and progesterone receptors.

**Case Presentation:**

In this article, we describe a case of a-69-year-old female with a painful mass in her left breast. Based on intraoperative pathology consult, neoplastic tissue mostly floating in mucinous lakes with invasion to surrounding stroma was seen. Immunohistochemistry profile showed positive estrogen and progesterone receptors and negative for HER2.

**Conclusion:**

Mucinous cystadenocarcinoma of breast is typically triple negative for hormone receptors. But ER and PR positive variant of this tumor is rare, giving the chance of a better prognosis for the patient with hormonal therapy.

Mucinous cystadenocarcinoma (MCA) is a relatively uncommon histological variant of breast cancer (1). It is composed of multiple cysts with more than 50% mucinous components (2, 3). It presents as a lobular gelatin-like and well defined mass on mammography, sonography and magnetic resonance imaging. It has a good prognosis because of the low incidence of axillary lymph node involvement and invasion to overlying skin. The short-term prognosis is very good especially when the tumor size is less than 5 cm. Mucinous cystadenocarcinoma is usually diagnosed in women that are older than 55-60 years. The incidence of MCA is about 1-6% of primary breast cancers (2, 4). On immunohistochemical stain, estrogen receptor (ER), progesterone receptor (PR) and HER2 are negative (5-7). Here, we present an uncommon case of MCA of left breast in a 69-year-old female which has a different IHC report. 

## Case presentation

A 69-year-old female referred to the clinic with a painful lump in her left breast 3 months ago. She also complained of bloody discharge from left nipple four years ago.

**Figure 1:**
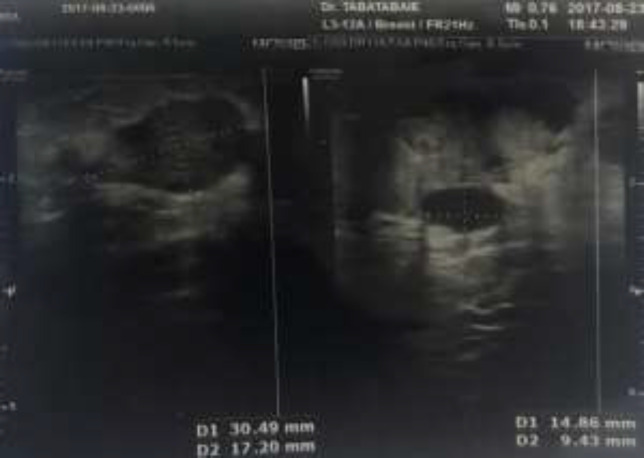
Sonography of left breast. A hypoecho mass with microcystic component was seen in retroareolar site

Despite recommendations for diagnostic procedure, the patient decided not to follow-up. On physical examination, an erythematous tender mass, owning hard consistency on palpation was detected around the nipple led to further radiological investigations. Sonography showed lobulated hypoecho mass with microcyctic areas measuring 20×17 mm at 3o’clock beside areol (Fig 1). Mammography also showed a hyperdense mass in retroareolar site. The patient was admitted and underwent surgery. According to the intraoperative pathology consultation based on multiple frozen sections from the mass, left modified radical mastectomy and lymph node dissection done. 

**Figure 2:**
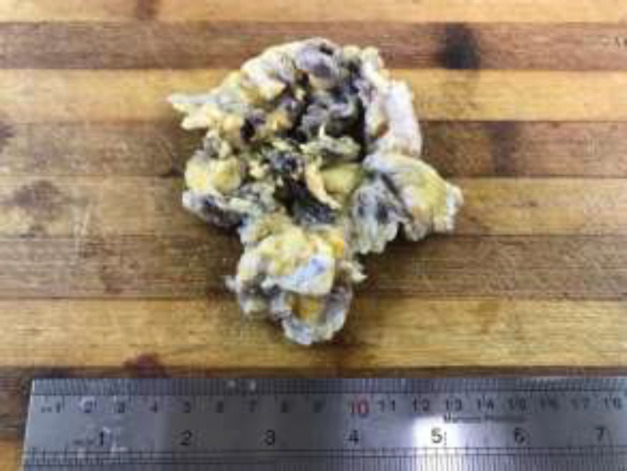
Gross picture shows tan-brown firm mass with multiple cyst formation

**Figure 3:**
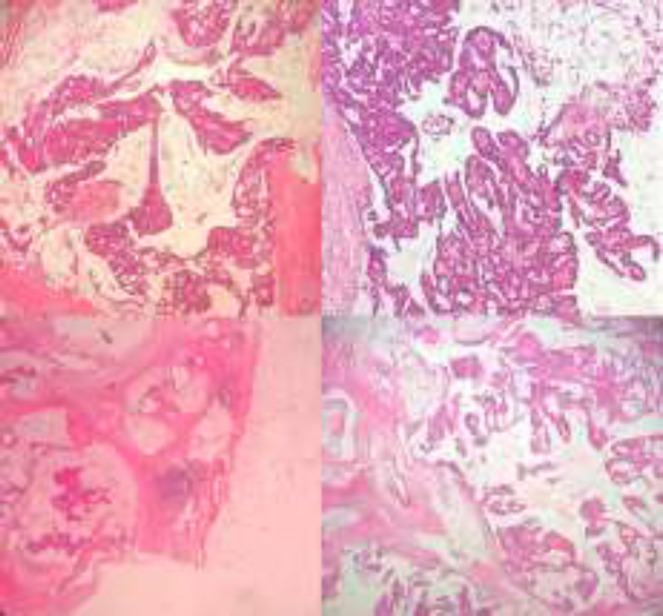
Microscopic sections show several cystic areas filled by mucinous secretion and lined by papillary and cribriform neoplasm

**Figure 4:**
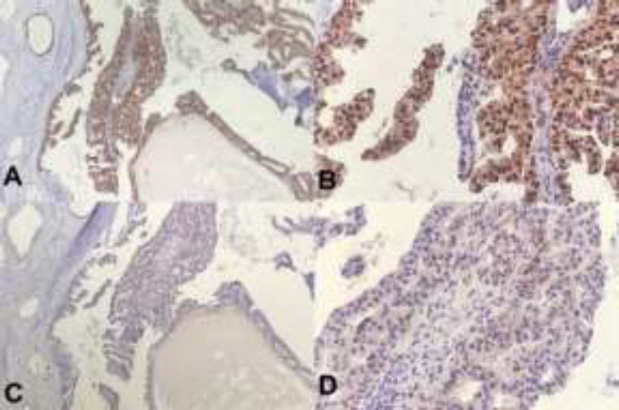
In immunohistochemistry staining for A) Estrogen Receptors (10×), B) Estrogen Receptors (40×); tumoral cells show moderate to intense nuclear positivity. C) Progesterone receptors (10×), D) Progesterone receptors (40×); tumoral cells show weak nuclear positivity

The specimen was received in formalin. On serial sectioning, there were tan-brown firm mass with multiple cystic areas. On gross examination, the surgical margins are free from tumoral cells and nipple was retracted (fig 2). Sections were taken and stained by hematoxylin and eosin. Microscopically, neoplastic tissue was seen, composed of dilated ducts, filling by papillary projections, cribriform and fused glands, lined by mild to moderate atypical cells with scattered mitoses and without distinct, myoepithelial layer mostly floating in mucinous lakes with invasion to surrounding stroma (figure 3). Foci of ductal carcinoma in situ including cribriform, papillary and micropapillary pattern were present in about 30% of tumor bulk. All surgical margins were free of tumor. Insitu component with papillary feature was seen in nipple. Nearby skin-deep dermis was involved by tumor. All of 16 lymph nodes showed reactive changes. There was no perineural or lymphovascular invasion. Immunohistochemistry profile showed positive estrogen and progesterone receptors and negative for HER2 (figure 4). The patient was finally discharged with regular follow-up.

## Discussion

Mucinous cystadenocarcinoma of the breast is a rare variant of breast carcinoma which has a higher incidence in peri- and post-menopausal women (8). Mucinous carcinoma typically has two subtypes, the cystic (mucinous cystadenocarcinoma) and solid (columnar cell mucinous carcinoma) (9). Microscopically, there are cystic spaces which are lined by mostly bland-looking columnar mucinous cells with some papillary formations. Nuclear atypia is evident in some foci, with some eosinophilic squamoid cell differentiations (10). On immunohistochemical stain, estrogen receptor (ER), progesterone receptor (PR), Her2neu are negative (5, 6). We report a case of a mucinous cystadenocarcinoma of the breast in addition to the characteristic immunophenotype ER (+), PR (+) and Her2 (-).

In comparison to intraductal carcinoma, hormone therapy is applied to pure mucinous carcinoma patients and chemotherapy is less useful for them, probably because of the higher ER or PR receptor expression and lower nuclear grade of MCA. Although mucinous cystadenocarcinomas are mostly negative for ER and PR (2). In our case because of positivity of hormone receptors which is so rare. hormonal therapy can be beneficial for the patient. Because of low incidence of lymph node involvement, mucinous cystadenocarcinoma has a relatively good prognosis (6). Despite the relatively enormous size of the tumor, the margins are free and no lymph node metastasis is identified hence rendering favorable prognosis. Careful clinical and radiological correlation and a review of patient’s prior medical history are needed to rule out metastasis from ovarian, pancreas or colorectal mucinous carcinoma, although the breast is the rarest area for metastasis from these regions (11).

In conclusions mucinous cystadenocarcinoma of breast typically is triple negative for hormonal receptors. It is important to know that ER and PR positive variants of this tumor is rare, giving the chance of a better prognosis for the patient with hormonal therapy.
